# Fine‐root decomposition characteristics of four typical shrubs in sandy areas of an arid and semiarid alpine region in western China

**DOI:** 10.1002/ece3.5133

**Published:** 2019-04-12

**Authors:** Ling‐Xianzi He, Zhi‐Qing Jia, Qing‐Xue Li, Li‐Li Feng, Kai‐Yue Yang

**Affiliations:** ^1^ Institute of Desertification Studies Chinese Academy of Forestry Beijing China; ^2^ Gonghe Desert Ecosystem Research Station Qinghai China

**Keywords:** Alpine sandy land, decomposition rate, fine‐root, Gonghe basin, nutrient release rate

## Abstract

**Background and aims:**

Research into the variability of fine‐root decomposition and nutrient cycling processes in arid and semiarid ecosystems is highly significant not only for investigations of regional and global carbon and nitrogen cycling but also for offering a theoretical basis for vegetation restoration and reconstruction. In particular, information is limited on fine‐root decomposition processes and nutrient releasing characteristics in the high‐altitude Qinghai Gonghe basin, which has different tree species and variable fine‐root diameters.

**Materials and methods:**

Four types of Salicaceae and Caragana shrubs were selected at the Qinghai Gonghe desert ecosystem research station. The litterbag method was adopted to measure decomposition rates of fine‐roots with three diameter classes (1–2 mm, 0.5–1 mm, and 0–0.5 mm). Chemical analysis was performed to determine nutrient (C, N, P, and K) concentrations of fine‐root, and nutrient release rates were compared among fine‐roots with different diameters during different decomposition periods. The differences in mass residual ratio and nutrient release rate among different diameter classes were studied with one‐way ANOVA.

**Results:**

Fine‐root decomposition rates were in the order *Caragana intermedia* > *Caragana korshinskii* > *Salix psammophila* > *Salix cheilophila*. Fine‐root decomposition showed a trend of “fast‐slow‐fast” variation, and decomposition rate increased as the diameter of fine‐roots increased, irrespective of tree species. During the decomposition process, the nutrients C, N, and P of fine‐root were in a release state for the four shrubs with different fine‐root diameters, and the corresponding release rates of Caragana shrubs were higher than those of Salicaceae shrubs. Release rates of nutrients C and N accelerated as fine‐root diameter increased, whereas release rates of nutrients P and K had no observed relation with fine‐root diameter. Fine‐root decomposition ratio was significantly correlated with initial values of N, P, C/N, C/P, and N/P of fine‐root. Fine‐root mass loss ratio was significantly correlated with initial concentration of soil nutrient K, and the correlation was positive for fine‐roots with diameters of 0–0.5 mm and 0.5–1 mm; however, no other significant correlation was observed between fine‐root mass loss ratio and initial soil environmental factors within this study.

**Conclusions:**

Our study showed that tree species and fine‐root diameter strongly affected decomposition rates, whereas diameter class exerted little effect on nutrient release rates.

## 1 INTRODUCTION

Desertification is widely recognized as a significant environmental problem, which is described as land degradation in arid, semiarid, and dry subhumid areas that is caused by a variety of factors, including climate variation and human activities (Allington & Valone, 2010). For vegetation in arid and semiarid areas, fine‐roots (≤2 mm in diameter) are primary pathways for water and nutrients (Ferguson & Nowak, 2011; McCormack, Eissenstat, Prasad, & Smithwick, 2013) and primary components of dead roots because of their fast turnover and high metabolic activity (the annual turnover rate of the whole root system is 10%, while that of the fine‐root system is 56%; Gaudinski et al., 2001). Some studies defined fine‐roots with diameter <1 mm (Thomas, Whitehead, & Reid, 1999), and also some scholars believed that roots with diameter <0.5 mm were more suitable to act as the diameter grade standard for fine‐roots (Pregitzer et al., 2002). Moreover, fine‐roots with diameter <2 mm usually have multiple grading orders. Fine‐root decomposition is one of the primary means for nutrient uptake and C exchange in terrestrial ecosystems (Burton, Pregitzer, & Hendrick, 2000). Although fine‐roots constitute a small proportion (3%–30%) of underground biomass, they possess 3%–84% of systematic primary productivity, which is consumed for fine‐root growth, respiration, and turnover (Gill & Jackson, 2000; Rytter, 2001; Yuan & Chen, 2010). The amount of C and N circulating through fine‐root production and decomposition is equal to or even greater than that of the aboveground counterpart (Fahey & Hughes, 1994), which implies that the circulation of nutrient elements in the soil would be underestimated by 20%–80% without consideration of the production, death, and decomposition of fine‐roots (Jackson, Mooney, & Schulze, 1997; Ruess et al., 2003; Steinaker & Wilson, 2005).

Fine‐root mass loss ratio (calculated as percentage of decreasing mass of fine‐roots occupying initial mass of fine‐roots) is affected by abiotic and biotic factors, including soil temperature, humidity, amount of substrate for fine‐root decomposition, and soil microbial and animal activities (Huang, Liao, Gao, Wang, & Yu, 2000; Lai et al., 2016). Silver and Miya (2001) stated that fine‐root decomposition rate was primarily influenced by the amount of fine‐root decomposition substrate, based on a statistical analysis of 175 fine‐root decomposition data from around the world, whereas the opposite was observed for 5 conifer species with different chronosequences in Oregon (Chen, Harmon, & Griffiths, 2001). Research shows that fine‐root decomposition substrate is primarily composed of lignin, C, N, and Ca (Chen et al., 2001; Hobbie, 2005; Lemma, Nilsson, Kleja, Olsson, & Knicker, 2007), among which an increase in N content had a positive effect on fine‐root decomposition (Goebel et al., 2011). Additionally, several studies show that fine‐root decomposition rate of broadleaf species is greater than that of conifer species, and that the rate is negatively correlated with fine‐root diameter (Camiré, Côté, & Brulotte, 1991; Tang et al., 2015; Wang, Cheng, Xiao, & Shen, 2017).

Many studies on fine‐root decomposition focused on temperate humid regions and low‐altitude areas (Parton et al., 2007; Wang et al., 2014; Xu et al., 2013), and these studies find that decomposition weakens over time in these relatively stable and favorable temperature–humidity conditions. However, information on fine‐root decomposition processes in high‐altitude arid and semiarid ecosystems is limited. The Gonghe basin, which is located in the northeastern part of the Tibet Plateau, belongs to the transitional region of Alpine arid desert and semiarid grassland in climate type. The Gonghe basin has been subjected to the most severe land desertification such that desertification land area occupies 91.9% of the total area, with typical characteristics of high altitude, low temperature, and short frost‐free period, compared with those of other arid and semiarid regions (Li, Jia, Liu, Feng, & He, 2017). Typical species for vegetation restoration in the Gonghe basin are *Salix cheilophila*, *Salix psammophila*, *Caragana intermedia*, and *Caragana korshinskii*, which play a significant role in water–soil conservation, as windbreaks and for sand fixation (Yu et al., 2015). Many investigations have focused on factors affecting belowground or aboveground litter decomposition and change in nutrients, including for *Sabina vulgaris*, *Artemisia ordosica*, *Caragana microphylla*, *Salix gordejevii*, *Artemisia halodendron*, *Salix psammophila,*and *Hedysarum mongolicum*, within Mu Us Sandy Land and Horqin Sandy Land (Lai et al., 2016; Qin, Hu, Wang, Na, & Zhang, 2010; Qu et al., 2010). However, few studies have been conducted on fine‐root decomposition in the Gonghe basin at high altitude and low temperature.

How fast do fine‐roots of four different types of vegetation decompose in a growing season or nongrowing season (relatively low temperature)? How will fine‐root mass loss ratio change when in another growing season? How can we describe the effect of diameter class on fine‐root mass loss and change in nutrient (C, N, P, and K) dynamics? To answer these questions, in this paper, four typical sand‐fixing species of vegetation, *S. cheilophila*, *S. psammophila*, *C. intermedia*, and *C. korshinskii*, were selected, which were planted in 1990 in the Gonghe basin. Their fine‐roots were sorted into three classes (1–2 mm, 0.5–1 mm, and 0–0.5 mm), and the effect of diameter class on fine‐root mass loss and nutrient (C, N, P, and K) dynamic change characteristics was determined, to obtain theoretical data on the belowground ecological cycle for desertification lands.

## 2 MATERIALS AND METHODS

### 2.1 Study site description

**Figure 1:**
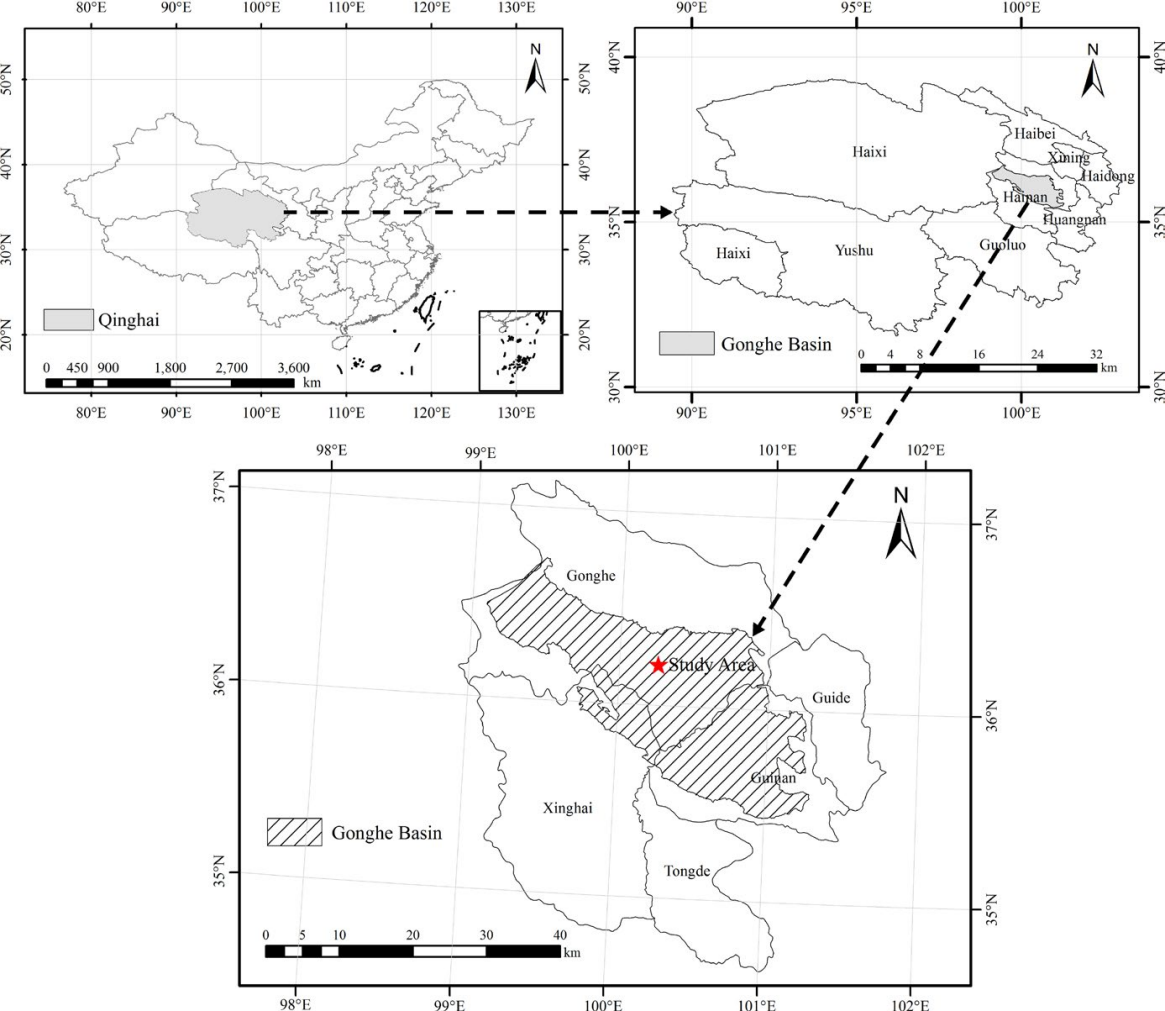
Location of the study area, Gonghe County, Qinghai Province, China

The study was conducted at the Qinghai Gonghe desert ecosystem research station located in the northeastern part of the Tibet Plateau (36°03–36°40′N, 99°45′–100°30′E; altitude 2,871–3,870 m), western China, as Figure 1 illustrates. The climate in the study area is a plateau continental climate with mean annual temperature, precipitation, and potential evaporation of 1.0–5.2°C, 311.1–402.1 mm, and 1716.7 mm, respectively. The mean annual number of windy days is 50.6 days (up to 97 days). The wind directions are primarily west and northwest, with a mean annual wind speed of 2.7 m/s (up to 40 m/s). Sand dune and dune slack were distributed alternately in the vegetation restoration area, and the primary geomorphic types were active dune, semiactive dune, fixed dune, semifixed dune, and dune slack. The zonal soils in the region were chestnut soil and brown calcic soil; whereas the nonzonal ones were eolian sandy soil and meadow soil (Li et al., 2015). Artificial vegetation is the primary vegetation type in the study area, which includes tree species (e.g., *Populus simonii*and *Populus cathayana*), shrub species (e.g., *C. intermedia, C. korshinskii, S. cheilophila,*and *S. psammophila*), and semishrub species (e.g., *A. ordosica*), whereas natural vegetation accounts for a small portion that includes *Caragana tibetica*, *Nitraria tangutorum*, *Achnatherum splendens*, and *Stipa* spp. (Li et al., 2017; Zhang, 2009).

### 2.2 fine‐root decomposition

Four types of *Salicaceae* shrubs (*S. cheilophila*and *S. psammophila*) and *Caragana* shrubs (*C. intermedia*and* C. korshinskii*) were selected near the Qinghai Gonghe desert ecosystem research station, which were planted in 1990 on dune slack and semifixed dune, respectively. The two types of *Salicaceae* shrubs were mixed in belts with plant spacing of 1 m * 2 m. Five rows of plants were in one belt for *S. cheilophila*, whereas *S. psammophila* were in three rows, with belt spacing of both at 4 m. The two types of *Caragana* shrubs were bunch planted in the center of a clay–sand barrier (1 m * 1 m) paved on active dune. Three 5 m * 5 m quadrats were established for *Salicaceae* and *Caragana* forests in May 2015, and sample information is shown in Table 1.

**Table 1:** Characteristics of sample plots

Plot information	*Salix cheilophila*	*Salix psammophila*	*Caragana intermedia*	*Caragana korshinskii*
Quadrat (m*m)	5*5	5*5	5*5	5*5
Altitude (m)	2,881	2,881	2,878	2,878
Biotope	Dune slack	Dune slack	Semifixed dune	semifixed dune
Forestation technique	Cuttage	Cuttage	Bunch planting, clay–sand barrier	bunch planting, clay–sand barrier
Accompanying species	*Potentilla chinensis*, *Leymus*, *Adsurgens*, *Aster*	*Alfalfa*, *Liquorice*	*Leymus, Sedge, Suaeda*	*Leymus, Sedge, Suaeda*
Mean height (m)	2.98 ± 0.06	2.74 ± 0.06	1.61 ± 0.03	2.21 ± 0.11
Plant crown (m*m)	4.20*4.04	3.05*2.84	1.97*2.02	2.26 * 2.18
Canopy coverage (%)	80	75	25	35
Soil type	Sandy soil (clay)	Sandy soil (clay)	Sandy soil	sandy soil
Soil bulk density (g cm^−3^)	1.51 ± 0.04	1.44 ± 0.01	1.42 ± 0.08	1.49 ± 0.06
Soil moisture (%)	7.85 ± 0.03	9.64 ± 0.02	2.45 ± 0.01	2.01 ± 0.01
Soil organic carbon (g kg^−1^)	7.24 ± 1.19	11.44 ± 1.35	4.01 ± 1.05	3.39 ± 0.93
Soil total N (g kg^−1^)	0.73 ± 0.12	1.11 ± 0.07	0.54 ± 0.09	0.34 ± 0.05
Soil total P (g kg^−1^)	0.61 ± 0.01	0.64 ± 0.03	0.46 ± 0.02	0.47 ± 0.05
Soil total K (g kg^−1^)	18.20 ± 0.81	18.71 ± 0.51	16.54 ± 0.28	16.46 ± 1.07

Notes

Soil from the upper 20–40 cm of the soil profile was analyzed in this study.

Values are expressed as the mean ± *SE*, with a replication number of five.

The root samples of the four types were obtained randomly from the surface soil layer (0–20 cm) by digging within quadrats, and then, dark, inelastic, and dead roots were eliminated in the laboratory. The buried litterbag method was adopted to measure fine‐root mass residual ratio. Litterbags (10 cm * 15 cm) were made of 0.5 mm nylon mesh. Fine‐roots were washed and air‐dried at room temperature to constant weight and sorted into three diameter classes (1–2 mm, 0.5–1 mm, and 0–0.5 mm; Smit, George, & Groenwold, 2000). The fine‐roots were cut into 2 cm lengths and mixed for each class, with each litterbag filled with a certain amount (2 g). Five plants that had a good growth state, and similar growth vigor were selected as standard plants below which the nylon litterbags were buried in 30‐cm deep holes. Fine‐roots of 1–2 mm, 0.5–1 mm, and 0–0.5 mm were put into the hole successively as one group, with an intragroup distance of 10 cm and intergroup distance of 20 cm. For each diameter class, 75 bags of fine‐roots were prepared and covered by forest soil and litter in the study area. The buried litterbags were removed in July and September in 2015 and June, August, and October in 2016 (15 bags each time for every diameter class every species). Because of low temperatures from November to the following April, the experiment was scarcely conducted. New roots were removed, and the rest of the roots in the litterbags were washed and air‐dried at room temperature to constant weight. The fine‐roots were prepared through milling and 100‐mesh sieving, and samples were used to measure nutrient (C, N, P, and K) contents for different diameter classes.

### 2.3 Determination of soil properties

Soil samples were obtained by soil auger 30 cm from the center of a standard plant in the four geographic directions and mixed evenly. Three points were sampled for each sample area, and three replications were performed. Soil samples were dried in the shade, sieved through a 100 mesh, and used for soil nutrient (C, N, P, and K) analyses. Undisturbed soil was also collected at the same sample depth using the cutting ring method for determination of soil bulk density. Soil samples were weighed before and after 12 hr of immersion and 105°C treatment in the oven, and soil volume weight, water content, and maximum water holding capacity were calculated.

### 2.4 Chemical analyses

Total C and N of fine‐roots were determined using an Elementar CHNS analyzer (Vario EL III, Elementar Analyser Systeme; GmbH, Germany); whereas total P and K were analyzed with the HNO_3_ digestion method using a 6300 ICP‐AES (Thermo Scientific, USA). Total N of soil was measured with the semimicro‐kjeldahl method using a kjeldahl apparatus (Kjeldahl 2200 Auto Distillation Unit; FOSS, Sweden); whereas total P and K concentrations were obtained with the HF‐HClO_4_‐HNO_3_ digestion method using a 6300 ICP‐AES (Thermo Scientific, USA).

### 2.5 Data analyses

The nonlinear exponential attenuation model (Olson, 1963) was introduced for regression analysis, and the relevant equations are presented below.

(1) $$y=X_{t}/X_{0}*100\%=\left(1-X_{s}/X_{0}\right)*100\%$$

(2) $$y=ae^{-kt}$$

(3) $$\text{NRT}=\left(X_{0}S_{0}-X_{t}S_{t}\right)/X_{0}S_{0}*100$$

(4) $$T_{50}=\ln\left(50/a\right)/\left(-k\right)$$

(5) $$T_{95}=\ln\left(5/a\right)/\left(-k\right)$$

For the above, *y* is the mass residual ratio of fine‐roots (%), *X*_t_ is fine‐root residual mass (g), *X*_s_ is the fine‐root loss mass (g), *X*_0_ is the initial mass (g), *a* is a fitting parameter, *t* is the time (*a* ^−1^), *k* is the decomposition coefficient (g g^−1^ a^−1^), *NRT* is the nutrient release rate (%), *S*_t_ is the remaining nutrient concentration (g g^−1^), *S*_0_ is the initial nutrient concentration (g g^−1^), and *T*_50_ and *T*_95_ are the times required for 50% and 95% decomposition (a), respectively.

All statistical analyses were conducted using the SPSS 19.0 statistical software package (SPSS Inc., Chicago, USA) with a significance level of *p* < 0.05, whereas figures were prepared with Origin 8.0 (OriginLab, Massachusetts, USA). The differences in C, N, P, and K initial concentrations among different species and diameter classes were analyzed with one‐way ANOVA and the LSD method, whereas the differences in mass residual ratio and nutrient release rates among different diameter classes were studied with one‐way ANOVA. Additionally, three‐way ANOVA was used to investigate the interaction effect of site condition, species, diameter class and decomposition time on fine‐root mass loss, and nutrient release rate. The correlations between mass loss ratio, nutrient content, and environmental factors of soil were also examined using Pearson correlation coefficient analysis.

## 3 RESULTS

### 3.1 fine‐root decomposition

**Figure 2:**
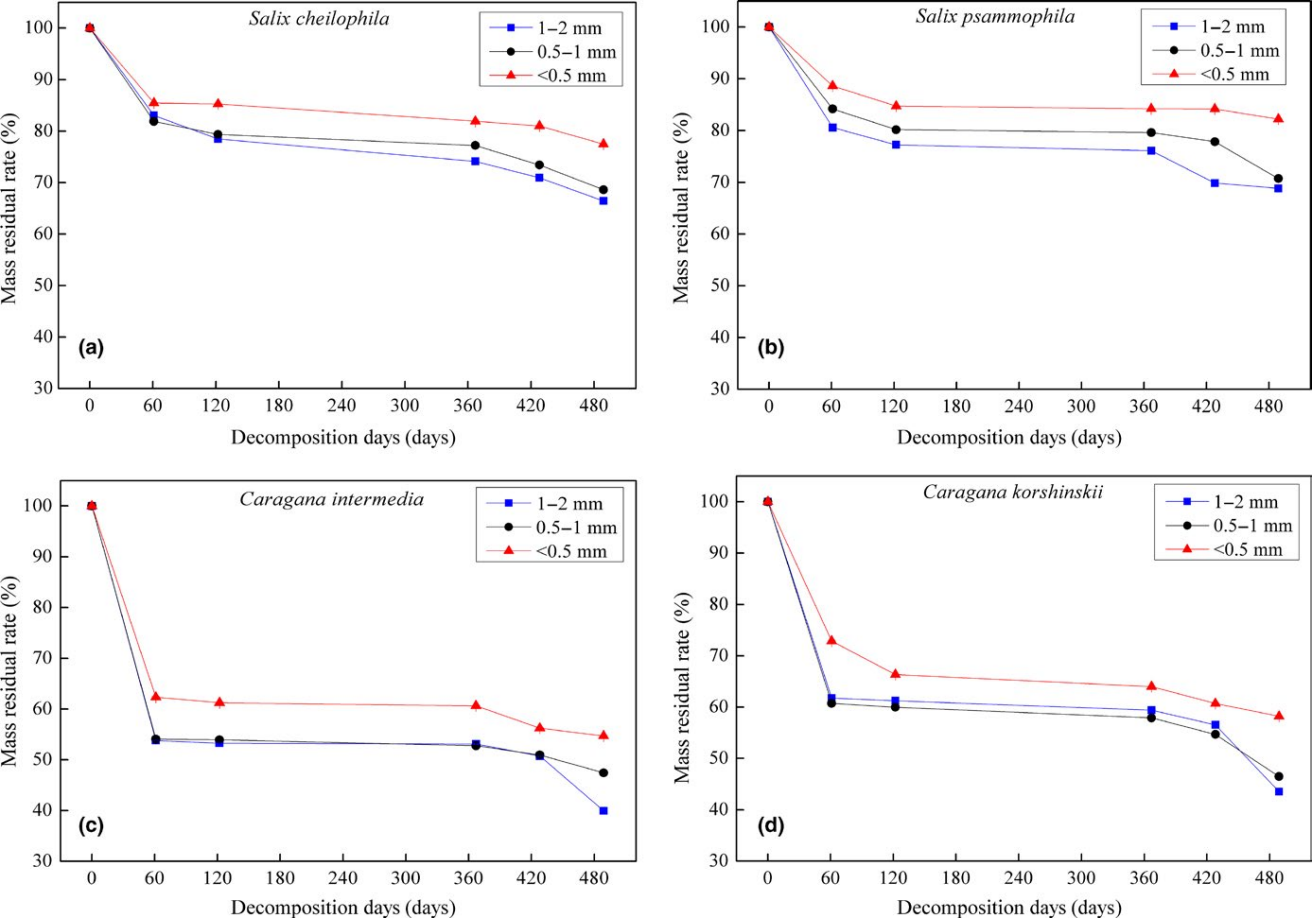
Mass residual ratio of fine‐roots with different diameters among four shrub species during 489 days of decomposition

Figure 2 shows the residual mass of fine‐roots with different diameters among the four shrub species during 489 days of decomposition. The fine‐root mass residual ratio was significantly different (*p* < 0.05) during the decomposition process. Fast decomposition occurred in the initial phase (0–120 days), and slow decomposition was observed in the second period (120–360 days), followed by obvious decomposition again in the last period (360–489 days).

During the initial period, fine‐root mass residual ratio was at the maximum for the first 60 days. As Figure 2 illustrates, the fine‐root mass residual ratio of *Caragana* shrubs planted in semifixed dune was greater than that of *Salicaceae* shrubs grown in dune slack. Mass residual ratio of fine‐roots was 53.28%–66.34% and 77.24%–85.26% for *Caragana* and *Salicaceae* shrubs after 120 days of decomposition, respectively, and the values were reduced to 39.94%–58.20% and 66.44%–82.23%, respectively, after 489 days of decomposition.

As shown in Table 2, fine‐root decomposition rate of the four sandy shrubs increased as the diameter increased. For the roots of the same diameter, decomposition rates of *Caragana* were distinctly greater than those of *Salicaceae* shrubs. Among the four shrubs, the time required for 50% and 95% decomposition was significantly different, which appeared as *S. psammophila* > *S. cheilophila* > *C. korshinskii* > *C. intermedia*.

**Table 2:** Olson exponential regression analysis of fine‐roots mass residual ratio for four species with different diameters

Species	Diameter (mm)	Decomposition coefficient (kg kg^−1^ a^−1^)	Measured value of 489 days residue (%)	Predicted value of 489 days residue (%)	*T*_50_ (a)	*T*_95_ (a)
*Salix cheilophila*	1–2	0.1595	66.438	68.281	3.29	17.73
	0.5–1	0.1012	68.603	71.164	4.25	23.23
	≤0.5	0.0733	77.463	78.999	7.58	39.00
*Salix psammophila*	1–2	0.1181	68.820	69.824	4.17	23.66
	0.5–1	0.0964	70.729	74.243	5.24	27.99
	≤0.5	0.0443	82.225	82.782	12.72	64.72
*Caragana intermedia*	1–2	0.2053	39.940	45.658	0.79	12.57
	0.5–1	0.1703	47.427	49.385	1.20	14.96
	≤0.5	0.1559	54.719	55.986	2.51	17.18
*Caragana korshinskii*	1–2	0.1647	43.548	50.211	1.36	14.77
	0.5–1	0.1213	46.495	50.846	1.44	26.41
	≤0.5	0.0881	58.200	59.076	2.61	27.35

### 3.2 fine‐root nutrient characteristics

#### 3.2.1 Initial nutrient contents

For the same species, no significant difference was observed in initial nutrient content between fine‐roots of 1–2 mm and 0.5–1 mm, which was different from that of the 0–0.5 mm group (Table 3). Initial C content and C/P ratio increased as the diameter increased, whereas initial P and K contents showed the opposite tendency. Moreover, N content variation showed two different processes. For initial N content and N/P ratio, the values were negatively correlated with diameter of fine‐roots for *S. cheilophila* and *S. psammophila*, whereas the values were positively correlated for *C. intermedia* and *C. korshinskii*. For the initial C/N ratio, the values increased with the increasing diameter of fine‐roots for *Salicaceae* shrubs in dune slack, whereas the values showed a “fall and rise” trend for *Caragana* shrubs in semifixed sandy dunes.

**Table 3:** Initial C, N, P, and K concentrations and their ratios for roots of four shrub species with three different diameters

Plot Information	Species	Diameter (mm)	C (g kg^−1^)	N (g kg^−1^)	P (g kg^−1^)	K (g kg^−1^)	C/N	C/P	N/P
Land among Sandy Dunes	*Salix cheilophila*	1–2	469.07 ± 5.27Aa	6.55 ± 0.18Db	1.81 ± 0.04Bb	5.64 ± 0.16Ca	71.68 ± 1.20Aa	258.89 ± 5.12Ba	3.61 ± 0.11Cb
		0.5–1	461.84 ± 8.73Aa	6.73 ± 0.23Db	1.88 ± 0.04Bb	5.75 ± 0.07Ca	68.72 ± 1.07Aa	246.14 ± 7.1Ba	3.59 ± 0.15Cb
		≤0.5	451.40 ± 3.46Aa	9.73 ± 0.46Ca	2.03 ± 0.03Bba	6.05 ± 0.22Ba	46.61 ± 2.23Ab	221.98 ± 3.78Bb	4.78 ± 0.15Ba
	*Salix psammophila*	1–2	478.63 ± 1.33Aa	8.42 ± 0.31Cb	2.06 ± 0.06Aa	5.21 ± 0.27Cb	57.00 ± 1.97Ba	232.64 ± 5.72Ba	4.08 ± 0.04Cb
		0.5–1	470.92 ± 1.16Aa	8.77 ± 0.09Cb	2.11 ± 0.09Aa	5.39 ± 0.11Cb	53.71 ± 0.68Ba	224.49 ± 9.63Ba	4.18 ± 0.13Cb
		≤0.5	434.78 ± 5.38Ab	10.94 ± 0.30Ca	2.27 ± 0.05Aa	6.86 ± 0.39Ba	39.82 ± 1.54Bb	192.11 ± 6.17Bb	4.83 ± 0.16Ba
Semifixed Sandy Dunes	*Caragana intermedia*	1–2	465.48 ± 5.59Aa	40.47 ± 0.82Aa	0.91 ± 0.02Cb	8.26 ± 0.01Ab	11.51 ± 0.25Ca	513.06 ± 11.42Aa	44.57 ± 0.05Aa
		0.5–1	462.40 ± 1.82Aa	40.26 ± 0.68Aa	0.96 ± 0.02Cab	9.01 ± 0.09Aa	11.49 ± 0.19Da	480.87 ± 11.23Aa	41.84 ± 0.32Ab
		≤0.5	412.43 ± 6.45Bb	33.56 ± 1.62Ab	1.04 ± 0.04Ca	9.19 ± 0.09Aa	12.33 ± 0.41Ca	398.80 ± 13.26Ab	32.36 ± 0.66Ac
	*Caragana korshinskii*	1–2	465.42 ± 3.27Aa	33.91 ± 0.44Ba	0.95 ± 0.04Ca	6.26 ± 0.14Ba	13.73 ± 0.25Ca	490.47 ± 26.08Aa	35.68 ± 1.38Ba
		0.5–1	459.59 ± 2.29Aa	33.71 ± 0.88Ba	0.96 ± 0.02Ca	6.54 ± 0.26Ba	13.65 ± 0.34Ca	481.42 ± 8.51Aa	35.32 ± 1.30Ba
		≤0.5	391.23 ± 6.31Cb	30.16 ± 0.63Bb	0.99 ± 0.04Ca	6.85 ± 0.17Ba	12.97 ± 0.08Ca	395.00 ± 15.91Ab	30.44 ± 1.13Ab

Notes

Different capital and lowercase letters represent significant differences among different species for the same fine‐root diameter and among different fine‐root diameters for the same species, respectively (*p* < 0.05).

Value are expressed as the mean ± *SE*, with a replication number of five.

For the same diameter class of fine‐roots, initial C and P contents and C/N ratio of *S. cheilophila* and *S. psammophila* were greater than those of *C. intermedia* and *C. korshinskii*; however, for the parameters N, K, C/P, and N/P, the results were the opposite.

**Figure 3:**
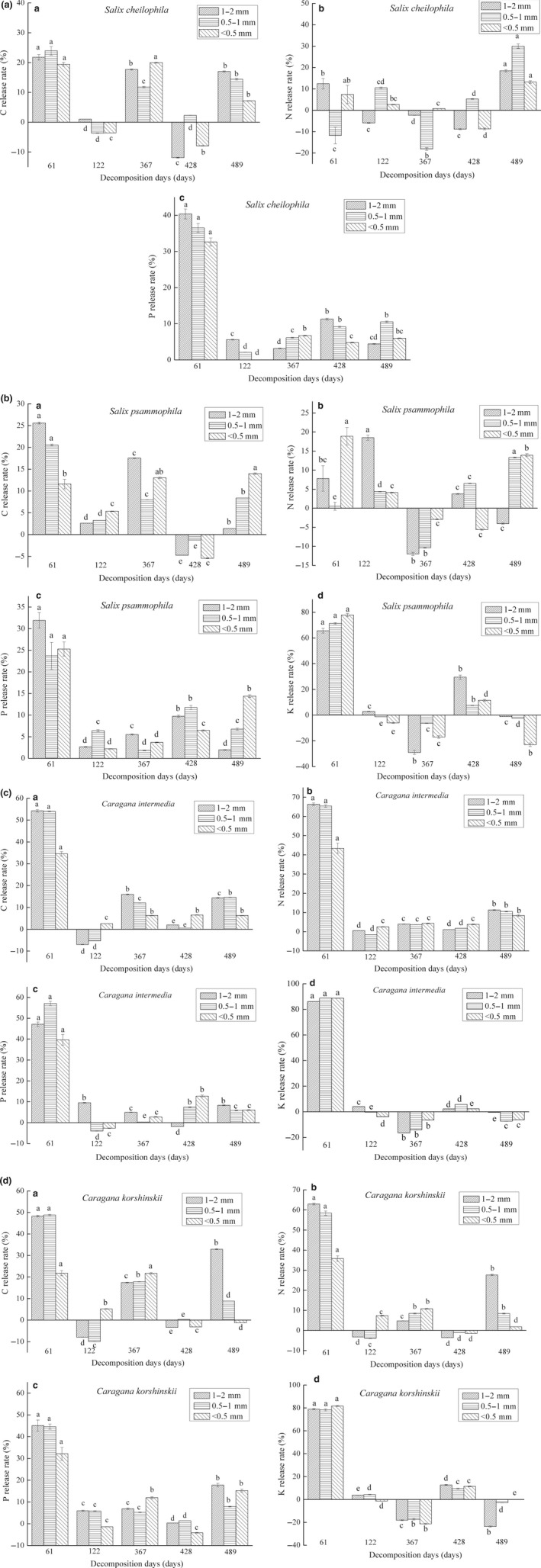
C, N, P, and K release rates of fine‐roots with different diameters among four shrub species

Figure 3 shows that different levels of release and enrichment of nutrients (C, N, P, and K) occurred during the process of fine‐root decomposition, and nutrient release rate accelerated as fine‐root diameter increased. Decomposition rate achieved the maximum during the first 60 days, and nutrient release rates of C and N were 21.87%–54.27% and 35.86%–66.43%, respectively, for *Caragana* shrubs, which were markedly larger than those for *Salicaceae* shrubs (11.63%–25.60% and 0.56%–19.02%, respectively). Next, the plants entered a mixed period of enrichment and release and then continued with nutrient release for the last period. After 489 days of decomposition, the release of nutrients C and N was 44.37%–87.39% and 54.41%–88.63%, respectively, for *Caragana* shrubs, whereas these values were 35.15%–48.86% and 14.00%–28.56%, respectively, for *Salicaceae* shrubs. Particularly, *C. intermedia*possessed the highest C and N release rates, whereas *S. psammophila*showed the lowest.

#### 3.2.2 Nutrient (C, N, P, and K) release rates

As shown in Table 4, synthetic effect of species, diameter class, and decomposition time on C release rate was not remarkable, whereas combined effect of species and time on N release rate was significant. The nutrient P was released in general, and the release rate varied among the four sandy shrubs. The nutrient K was released in the periods of 0–60 days and 367–428 days, whereas enrichment occurred in the other periods to different extents.

**Table 4:** Three‐way ANOVA for mass loss ratio and C, N, P, and K release rates

Variation source	*df*	Mass loss ratio		C release rate		N release rate		P release rate		K release rate	
		F	P	F	P	F	P	F	P	F	P
*S*	3	806.606	<0.001	3.414	0.034	7.204	0.001	1.108	0.365	0.468	0.708
*D*	2	135.18	<0.001	1.998	0.158	0.213	0.81	1.339	0.281	0.215	0.808
*T*	4	82.621	<0.001	55.654	<0.001	24.33	<0.001	122.743	<0.001	357.341	<0.001
*S* × *D*	6	1.596	0.192	0.392	0.877	0.297	0.932	0.255	0.952	0.463	0.829
*S* × *T*	12	1.702	0.129	3.297	0.006	6.3	<0.001	3.776	0.003	2.05	0.065
*D* × *T*	8	2.558	0.036	2.454	0.042	0.748	0.65	1.555	0.191	0.858	0.564

Note

*S*, *D* and *T* represent tree species, diameter size, and time, respectively.

The nutrient release rate affected the C/N ratio of the four sandy shrubs in the order *S. psammophila* > *S. cheilophila* > *C. korshinskii* > *C. intermedia*. The C/N ratio decreased during entire decomposition process of fine‐roots for *S. psammophila* and *S. cheilophila*, whereas the value increased during the first 60 day period and then experienced a mild decline for *C. korshinskii* and *C. intermedia*. Moreover, the C/N ratio increased with the increasing diameter of fine‐roots for the same tree species.

### 3.3 Influencing factors on fine‐root decomposition

#### 3.3.1 Relations between fine‐root mass loss ratio and initial nutrient concentrations

Pearson correlation analysis was performed between fine‐root mass loss ratio and initial nutrient concentrations for the four sandy shrub roots of different diameters. As shown in Table 5, fine‐root mass loss ratio was significantly positively correlated with initial values of N, C/P, and N/P, whereas the correlations with P and C/N were negative. The correlation was positive and significant between fine‐root mass loss ratio and initial amount of K for 428 days, whereas the correlation was not significant for the rest of the decomposition process. Additionally, no significant relation was found between fine‐root mass loss ratio and the initial concentration of C.

**Table 5:** Correlation coefficients between fine‐root mass loss ratio and initial nutrient concentrations for four shrub species

Time (d)	C	N	P	K	C/N	C/P	N/P
61	−0.034	0.957**	−0.931**	0.731**	−0.832**	0.970**	0.967**
122	0.046	0.922**	−0.913**	0.680*	−0.770**	0.957**	0.938**
367	0.147	0.724**	−0.582*	0.662*	−0.639*	0.706*	0.719**
428	0.085	0.920**	−0.956**	0.652*	−0.776**	0.958**	0.938**
489	0.041	0.900**	−0.938*	0.545	−0.749**	0.976**	0.927**

* Significant difference (*p* < 0.05) and

** Extremely significant difference (*p* < 0.01), respectively.

#### 3.3.2 Relations between fine‐root mass loss ratio and nutrient contents during the decomposition process

As shown in Table 6, fine‐root mass loss ratio was extremely significantly correlated with the amount of C, N, and P, with K the exception, for fine‐roots of diameter 1–2 mm and 0.5–1 mm, whereas for the diameter 0–0.5 mm, the correlations were extremely significant for the values of N and P but not for those of C and K.

**Table 6:** Correlation coefficients between fine‐root mass loss ratio and nutrient contents of four shrub species

Nutrients	Diameter (mm)		
	1–2	0.5–1	≤0.5
C	−0.683**	−0.654**	−0.312
N	0.666**	0.851**	0.900**
P	−0.844**	−0.926**	−0.917**
K	0.223	0.19	0.15

** Extremely significant difference (*p* < 0.01).

Regardless of diameter class, fine‐root mass loss ratio was related to the concentration of N positively and to that of P negatively, whereas little significant correlation existed for K. Additionally, as fine‐root diameter varied from 1–2 mm and 0.5–1 mm to 0–0.5 mm, the content variation of C decreased with diameter.

#### 3.3.3 Relations between fine‐root mass loss ratio and initial soil environmental factors

As shown in Table 7, fine‐root mass loss ratio was significantly positively correlated with initial content of soil K for fine‐roots of diameters 0–0.5 mm and 0.5–1 mm. However, no other significant correlation was observed between fine‐root mass loss ratio and initial soil environmental factor within this study.

**Table 7:** Correlation coefficients between fine‐root mass loss ratio and initial soil environmental factors of four shrub species

Soil environmental factors	Diameter (mm)		
	1–2	0.5–1	≤0.5
Soil water content	−0.249	−0.197	−0.185
Soil bulk density	−0.325	−0.307	−0.296
C	−0.283	−0.188	−0.148
N	−0.284	−0.202	−0.108
P	−0.531	−0.456	−0.426
K	0.533	0.620*	0.596*

* Significant difference (*p* < 0.05).

## 4 DISCUSSION

Fine‐root decomposition is a process of substance exchange with the external environment in which soil biological metabolism occurs with the absorption and release of chemical elements upon the action of soil leaching and breakdown (Burton et al., 2000). In the early stage of decomposition, rapid eluviation of carbohydrates and other soluble substances is caused by environmental factors, such as soil temperature and soil moisture content (Parton et al., 2007; Tu, Su, Zhang, Fan, & Zhou, 2014) and the decomposition substrate. In the later period, the decomposition process is primarily affected by the biological action that exhausted the soluble compounds, with insoluble substances (lignin and cellulose, among others) remaining for slow microbiological degradation.

In this study, mass residual ratio was 15%–47% after the first 120 days of decomposition for the four sandy shrubs; whereas the ratio was 0.5%–3% during the period from 120 to 360 days. Similarly, mass residual ratio was 15%–25% after the first 150 days of fine‐root decomposition for *A. ordosica*, *S. psammophila,* and *H. mongolicum* planted in the Mu Us Desert, whereas the value remained at only 5%–10% during the following period of 2 years (Lai et al., 2016). Therefore, the long‐term effect of fine‐root decomposition on the environment was poorly reflected and would be difficult to be predicted accurately based on the short‐term decomposition process, which indicated that the range of decomposition periods should be arranged more rationally in further studies.

Two types of *Reaumuria songarica* planted in the desert area in Xinjiang possessed a *T*_95_ of 6.13–8.04 a (Zhao et al., 2014). For *C. korshinskii* and *S. psammophila* planted on Mu Us sand land in a semiarid region in Ningxia, the value of *T*_95_ was 8.52 a and 18.80 a, respectively (Lai, 2015), which are values smaller than the corresponding data obtained within this study. This case might result from climate and soil temperature. The current study was conducted in an alpine sandy land in Qinghai at an altitude of 2,871–3,870 m, which was greater than that of the experimental fields of Lai and Zhao (Ningxia and Xinjiang, respectively). Soil temperature was relatively low; thus, microbial activities and the activity of degradative enzymes weakened markedly, which further slowed the decomposition rate and increased the decomposition time. Therefore, fine‐root decomposition rate differed distinctly even for the same species as the study area varied.

As presented in Figure 3, the N release rate of *C. intermedia* and *C. korshinskii* was greater than that of *S. cheilophila* and *S. psammophila*, which might be attributed to the natural nitrogen fixation of *Caragana* plants. As in other leguminous plants, nodule bacteria parasitic on roots of *C. intermedia* and *C. korshinskii* may increase soil microbial decomposition activity without the necessity of N absorption from the external environment, because the roots themselves provide sufficient N (Manzoni, Jackson, Trofymow, & Porporato, 2008). The relatively high N concentration of fine‐roots accelerated microbial decomposition activity, which led to faster fine‐root decomposition.

Significant positive correlation was observed between fine‐root mass loss ratio and the content variation of N (Table 6). Similar research has been conducted with other plants. Tu et al. (2015) studied fine‐root decomposition of *Pleioblastus amarus* in a Sichuan subtropical forest and concluded that decomposition rate was affected by internal (kept inside vegetations) and external (obtained due to external factors) N concentrations. In some other studies, fine‐root decomposition rate depended primarily on the amount of lignin and initial C rather than initial N concentration (Chen et al., 2001), which is a result not consistent with the findings in this study. This case might occur from the restraint on lignin decomposition with soil temperatures and water content reduced dramatically, and nutrient enrichment of N occurred in the late period of fine‐root decomposition. Additionally, the threshold value of the C/N ratio differed greatly because of the variety of tree species and environmental factors in the study area, which caused further differences in correlations between the fine‐root decomposition process and nutrient (C and N) dynamic variation process.

Synthesis of Table 3 and Figure 3 revealed that the nutrient P was in a release state overall during the fine‐root decomposition process, and fine‐root mass loss ratio was significantly negatively correlated with P concentration of fine‐roots irrespective of diameter class. A similar trend was also observed for *P. amarus* in the subtropical zone in Sichuan and seven types of evergreen tree species planted in the Fujian Jian'ou subtropical forest (Lin, Yang, Guo, Chen, & Xie, 2011; Tu et al., 2015). A possible reason for this finding was that microbial decomposition is supported by continuous release of fine‐root nutrient P itself, based on the condition of high initial P concentration inside fine‐roots and low P concentration in the soil within the research region in Gonghe County, Qinghai Province.

The fine‐roots with the largest diameter had the highest mass residual ratio (Figure 2). As fine‐root diameter increases, nutrient C is more likely to exist in the form of carbohydrates, amino acids, and other easily decomposable substances, which contribute to fast decomposition of fine‐roots (Chapin et al., 2000).

Diameter class exerted little effect on fine‐root nutrient (N, P and K) release rates. In other research, the release of a nutrient element to the environment is more likely with a high initial nutrient concentration, whereas nutrient enrichment occurs when a nutrient element is at a low initial concentration (Vogt, Grier, & Vogt, 1986).

Fine‐root mass loss ratio was significantly positively correlated with the initial concentration of soil nutrient K for fine‐roots of diameters 0–0.5 mm and 0.5–1 mm, based on correlation analysis data presented in Table 7. This case might occur because of the effect of the initial amount of soil K on fine‐root chemical properties, further affecting fine‐root decomposition rate (Prescott, 2005).

Fine‐root decomposition and nutrient cycling in arid and semiarid ecosystems is necessary for regional investigations of carbon and nitrogen cycling as well as for offering a theoretical basis for vegetation restoration. However, how the fine‐root decomposition can be used to gather estimates C cycling in these regions. A possible explanation could be that in addition to dead fine‐root, insoluble organic matter from roots, refractory microbial biomass, and live root exudates mostly contribute to SOC (soil organic carbon) accumulation at deeper soil depths in some regions (Matamala, Gonzàlez‐Meler, Jastrow, Norby, & Schlesinger, 2003; Uselman, Qualls, & Lilienfein, 2007). Several studies have mentioned the importance of SOC originating from live roots as root exudates (Uselman et al., 2007). These results indicated that the fine‐root litter of different shrub plots contributes to SOC accumulation at different rates and the mechanisms of SOC accumulation varies among shrub plots. An increase in SOC stocks mainly depends on the rate of fine‐root decomposition, which is affected by genetic and environmental factors (Hobbie, Oleksyn, Eissenstat, & Reich, 2010). Some studies demonstrated that fine‐root decomposition was important for C input to soil (Hobbie et al., 2010; Lemma et al., 2007). SOC stocks have an important impact on regional carbon cycle, and also have an important guiding significance for plantation area, planting method, and planting density of plantations.

## 5 CONCLUSIONS

The fine‐root decomposition process and nutrient release characteristics were studied for four typical sandy shrubs, *S. cheilophila*, *S. psammophila*, *C. intermedia*, and *C. korshinskii*, planted on alpine sandy land in the Gonghe basin. Decomposition rate differed markedly among different species. During 489 days of the decomposition process, *C. intermedia* possessed the highest fine‐root decomposition rate followed by *C. korshinskii*, *S. psammophila,* and *S. cheilophila* in sequence. Fine‐root decomposition showed a trend of “fast‐slow‐fast” variation, with the fastest decomposition occurring in the initial period of 120 days. Regardless of tree species, the decomposition rate increased as the diameter of fine‐roots increased. The nutrients C, N, and P were in a release state for the four shrubs with different fine‐root diameters, and the corresponding release rates of *Caragana* shrubs were higher than those of *Salicaceae* shrubs. The release rates of the nutrients C and N accelerated as fine‐root diameter increased, whereas no relation was observed between the release rates of P and K and fine‐root diameter. More attention should be directed to the relations between fine‐root decomposition rate and dynamic change of various environmental factors in future studies, for intensive investigation of soil nutrient cycling mechanisms in alpine regions.

## CONFLICT OF INTEREST

There are no relevant conflicts of interest.

## AUTHOR CONTRIBUTIONS

Ling‐Xianzi He and Zhi‐Qing Jia contributed to the conception of the study; Ling‐Xianzi He, Qing‐Xue Li and Li‐Li Feng contributed significantly to analysis and manuscript preparation; Ling‐Xianzi He performed the data analyses and wrote the manuscript; Kai‐Yue Yang helped perform the analysis with constructive discussions.

## Data Availability

All authors agree to archive the data of the article into any publicly accessible database.

## References

[ece35133-bib-0001] Allington, G. R. H. , & Valone, T. J. (2010). Reversal of desertification: The role of physical and chemical soil properties. Journal of Arid Environments, 74(8), 973–977.

[ece35133-bib-0002] Burton, A. J. , Pregitzer, K. S. , & Hendrick, R. L. (2000). Relationships between fine root dynamics and nitrogen availability in Michigan northern hardwood forests. Oecologia, 125(3), 389–399. 10.1007/s004420000455 28547334

[ece35133-bib-0003] Camiré, C. , Côté, B. , & Brulotte, S. (1991). Decomposition of roots of black alder and hybrid poplar in short‐rotation plantings: Nitrogen and lignin control. Plant and Soil, 138(1), 123–132. 10.1007/BF00011814

[ece35133-bib-0004] Chapin III, F. S. , Zavaleta, E. S. , Eviner, V. T. , Naylor, R. L. , Vitousek, P. M. , Reynolds, H. L. , … Díaz, S. (2000). Consequences of changing biodiversity. Nature, 405(6783), 234–243. 10.1038/35012241 10821284

[ece35133-bib-0005] Chen, H. , Harmon, M. E. , & Griffiths, R. P. (2001). Decomposition and nitrogen release from decomposing woody roots in coniferous forests of the Pacific Northwest: A chronosequence approach. Canadian Journal of Forest Research, 31(2), 246–260. 10.1139/x00-167

[ece35133-bib-0006] Fahey, T. J. , & Hughes, J. W. (1994). Fine root dynamics in a northern hardwood forest ecosystem, Hubbard Brook Experimental Forest, NH. Journal of Ecology, 82(3), 533–548.

[ece35133-bib-0007] Ferguson, S. D. , & Nowak, R. S. (2011). Transitory effects of elevated atmospheric CO_2_ on fine root dynamics in an arid ecosystem do not increase long‐term soil carbon input from fine root litter. New Phytologist, 190(4), 953–967. 21355868 10.1111/j.1469-8137.2011.03654.x

[ece35133-bib-0008] Gaudinski, J. , Trumbore, S. , Davidson, E. , Cook, A. , Markewitz, D. , & Richter, D. (2001). The age of fine‐root carbon in three forests of the eastern United States measured by radiocarbon. Oecologia, 129, 420–429. 10.1007/s004420100746 28547197

[ece35133-bib-0009] Gill, R. A. , & Jackson, R. B. (2000). Global patterns of root turnover for terrestrial ecosystems. New Phytologist, 147(1), 13–31.

[ece35133-bib-0010] Goebel, M. , Hobbie, S. E. , Bulaj, B. , Zadworny, M. , Archibald, D. D. , Oleksyn, J. , … Eissenstat, D. M. (2011). Decomposition of the finest root branching orders: Linking belowground dynamics to fine‐root function and structure. Ecological Monographs, 81(1), 89–102. 10.1890/09-2390.1

[ece35133-bib-0011] Hobbie, S. E. (2005). Contrasting effects of substrate and fertilizer nitrogen on the early stages of litter decomposition. Ecosystems, 8(6), 644–656. 10.1007/s10021-003-0110-7

[ece35133-bib-0012] Hobbie, S. E. , Oleksyn, J. , Eissenstat, D. M. , & Reich, P. B. (2010). Fine root decomposition rates do not mirror those of leaf litter among temperate tree species. Oecologia, 162(2), 505–513. 10.1007/s00442-009-1479-6 19882174

[ece35133-bib-0013] Huang, Z. , Liao, L. , Gao, H. , Wang, S. , & Yu, X. (2000). Decomposition process of Chinese fir stump roots and changes of nutrient concentration. Journal of Applied Ecology, 11(1), 40–42. 11766585

[ece35133-bib-0014] Jackson, R. B. , Mooney, H. A. , & Schulze, E. D. (1997). A global budget for fine root biomass, surface area, and nutrient contents. Proceedings of the National Academy of Sciences of the United States of America, 94, 7362–7366. 10.1073/pnas.94.14.7362 11038557 PMC23826

[ece35133-bib-0015] Lai, Z. (2015). Fine root dynamics of four typical Xerophilous shrubs and their effects on soil organic carbon. Beijing Forestry University, PhD Dissertation: 64–72.

[ece35133-bib-0016] Lai, Z. , Zhang, Y. , Liu, J. , Wu, B. , Qin, S. , & Fa, K. (2016). Fine‐root distribution, production, decomposition, and effect on soil organic carbon of three revegetation shrub species in northwest China. Forest Ecology and Management, 359, 381–388. 10.1016/j.foreco.2015.04.025

[ece35133-bib-0017] Lemma, B. , Nilsson, I. , Kleja, D. B. , Olsson, M. , & Knicker, H. (2007). Decomposition and substrate quality of leaf litters and fine roots from three exotic plantations and a native forest in the southwestern highlands of Ethiopia. Soil Biology and Biochemistry, 39(9), 2317–2328. 10.1016/j.soilbio.2007.03.032

[ece35133-bib-0018] Li, Q. , Jia, Z. , Liu, T. , Feng, L. , & He, L. (2017). Effects of different plantation types on soil properties after vegetation restoration in an alpine sandy land on the Tibetan Plateau, China. Journal of Arid Land, 9(2), 200–209. 10.1007/s40333-017-0006-6

[ece35133-bib-0019] Li, Q. , Jia, Z. , Zhu, Y. , Wang, Y. , Li, H. , Yang, D. , & Zhao, X. (2015). Spatial heterogeneity of soil nutrients after the establishment of *Caragana intermedia* plantation on Sand Dunes in Alpine Sandy Land of the Tibet Plateau. PloS One, 10(5), e0124456 10.1371/journal.pone.0124456 25946170 PMC4422674

[ece35133-bib-0020] Lin, C. , Yang, Y. , Guo, J. , Chen, G. , & Xie, J. (2011). Fine root decomposition of evergreen broadleaved and coniferous tree species in mid‐subtropical China: Dynamics of dry mass, nutrient and organic fractions. Plant and Soil, 338(1), 311–327. 10.1007/s11104-010-0547-3

[ece35133-bib-0021] Manzoni, S. , Jackson, R. B. , Trofymow, J. A. , & Porporato, A. (2008). The global stoichiometry of litter nitrogen mineralization. Science, 321(5889), 684–686. 10.1126/science.1159792 18669860

[ece35133-bib-0022] Matamala, R. , Gonzàlez‐Meler, M. , Jastrow, J. D. , Norby, R. J. , & Schlesinger, W. H. (2003). Impacts of fine root turnover on forest NPP and soil C sequestration potential. Science, 302, 1385–1387. 10.1126/science.1089543 14631037

[ece35133-bib-0023] McCormack, M. L. , Eissenstat, D. M. , Prasad, A. M. , & Smithwick, E. A. (2013). Regional scale patterns of fine root lifespan and turnover under current and future climate. Global Change Biology, 19(6), 1697–1708. 10.1111/gcb.12163 23504802

[ece35133-bib-0024] Olson, J. S. (1963). Energy storage and the balance of producers and decomposers in ecological systems. Ecology, 44(2), 322–331. 10.2307/1932179

[ece35133-bib-0025] Parton, W. , Silver, W. L. , Burke, I. C. , Grassens, L. , Harmon, M. E. , Currie, W. S. , … Fasth, B. (2007). Global‐scale similarities in nitrogen release patterns during long‐term decomposition. Science, 315(5810), 361–364. 10.1126/science.1134853 17234944

[ece35133-bib-0026] Pregitzer, K. S. , DeForest, J. L. , Burton, A. J. , Allen, M. F. , Ruess, R. W. , & Hendrick, R. L. (2002). Fine root architecture of nine North American trees. Ecological Monographs, 72(2), 293–309. 10.1890/0012-9615(2002)072]0293:FRAONN\2.0.CO;2.

[ece35133-bib-0027] Prescott, C. E. (2005). Do rates of litter decomposition tell us anything we really need to know. Forest Ecology and Management, 220(1), 66–74. 10.1016/j.foreco.2005.08.005

[ece35133-bib-0028] Qin, Y. , Hu, Y. N. , Wang, L. H. , Na, R. S. , & Zhang, G. S. (2010). Change of nutrient and energy in process of fine roots decomposition of Sabina vulgaris and Artemisia ordosica in Mu Us Sandland. Journal of Desert Research, 30(6), 1341–1347.

[ece35133-bib-0029] Qu, H. , Zhao, X. Y. , Zhao, H. L. , Wang, S. K. , Li, Y. Q. , & Liu, Z. G. (2010). Litter decomposition rates of three shrub species in Horqin Sandy Land and their relationship with key meteorological factors. Journal of Desert Research, 30(4), 844–849.

[ece35133-bib-0030] Ruess, R. W. , Hendrick, R. L. , Burton, A. J. , Pregitzer, K. S. , Sveinbjornssön, B. , Allen, M. F. , & Maurer, G. E. (2003). Coupling fine root dynamics with ecosystem carbon cycling in black spruce forests of interior Alaska. Ecological Monographs, 73, 643–662. 10.1890/02-4032

[ece35133-bib-0031] Rytter, R. M. (2001). Biomass production and allocation, including fine‐root turnover, and annual N uptake in lysimeter‐grown basket willows. Forest Ecology and Management, 140(2), 177–192. 10.1016/S0378-1127(00)00319-4

[ece35133-bib-0032] Silver, W. L. , & Miya, R. K. (2001). Global patterns in root decomposition: Comparisons of climate and litter quality effects. Oecologia, 129(3), 407–419. 28547196 10.1007/s004420100740

[ece35133-bib-0033] Smit, A. L. , George, E. , & Groenwold, J. (2000). Root observations and measurements at (transparent) interfaces with soil In Root methods (pp. 235–271). Berlin, Germany: Springer.

[ece35133-bib-0034] Steinaker, D. F. , & Wilson, S. D. (2005). Belowground litter contributions to nitrogen cycling at a northern grassland‐forest boundary. Ecology, 86(10), 2825–2833.

[ece35133-bib-0035] Tang, S. S. , Yang, W. Q. , Wang, H. P. , Xiong, L. , Nie, F. Y. , & Xu, Z. F. (2015). Decomposition and nutrient release of root with different diameters of three subalpine dominant trees in western area of Sichuan Province China. Chinese Journal of Applied Ecology, 26(10), 2921–2927. 26995898

[ece35133-bib-0036] Thomas, S. M. , Whitehead, D. , & Reid, J. F. B. (1999). Growth, loss, and vertical distribution of *Pinus radiata* fine roots growing at ambient and elevated CO_2_ concentration. Global Change Biology, 5(1), 107–121.

[ece35133-bib-0037] Tu, L. H. , Peng, Y. , Chen, G. , Hu, H. L. , Xiao, Y. L. , Hu, T. X. , … Tang, Y. (2015). Direct and indirect effects of nitrogen additions on fine root decomposition in a subtropical bamboo forest. Plant and Soil, 389(1), 273–288.

[ece35133-bib-0038] Tu, D. , Su, X. , Zhang, T. , Fan, W. , & Zhou, Q. (2014). Thermo-mechanical densification of Populus tomentosa var. tomentosa with low moisture content. BioResources, 9(3), 3846–3856.

[ece35133-bib-0039] Uselman, S. M. , Qualls, R. G. , & Lilienfein, J. (2007). Contribution of root vs. leaf litter to dissolved organic carbon leaching through soil. Soil Science Society of America Journal, 71(5), 1555–1563. 10.2136/sssaj2006.0386

[ece35133-bib-0040] Vogt, K. A. , Grier, C. C. , & Vogt, D. J. (1986). Production, turnover, and nutrient dynamics of above‐and belowground detritus of world forests. Advances in Ecological Research, 15, 303–377.

[ece35133-bib-0041] Wang, N. , Cheng, R. M. , Xiao, W. F. , & Shen, Y. F. (2017). Dynamics of fine root decomposition and its factors of *Pinus massoniana* in the Three Gorges Reservoir Area, China. Chinese Journal of Applied Ecology, 28(2), 391–398. 29749145 10.13287/j.1001-9332.201702.017

[ece35133-bib-0042] Wang, W. , Zhang, X. , Tao, N. , Ao, D. , Zeng, W. , Qian, Y. , & Zeng, H. (2014). Effects of litter types, microsite and root diameters on litter decomposition in *Pinus sylvestris* plantations of northern China. Plant and Soil, 374(1), 677–688. 10.1007/s11104-013-1902-y

[ece35133-bib-0043] Xu, W. , Liu, J. , Liu, X. , Li, K. , Zhang, D. , & Yan, J. (2013). Fine root production, turnover, and decomposition in a fast‐growth *Eucalyptus urophylla* plantation in southern China. Journal of Soils and Sediments, 13(7), 1150–1160. 10.1007/s11368-013-0718-y

[ece35133-bib-0044] Yu, Y. , Jia, Z. Q. , Zhu, Y. J. , Liu, Y. S. , Liu, H. T. , & Li, Q. X. (2015). Changes of carbon pools of alpine sandy *Salix cheilophila* shelterbelts with stand age. Acta Ecologica Sinica, 35(6), 1752–1760.

[ece35133-bib-0045] Yuan, Z. Y. , & Chen, H. Y. H. (2010). Fine root biomass, production, turnover rates, and nutrient contents in boreal forest ecosystems in relation to species, climate, fertility, and stand age: Literature review and meta‐analyses. Critical Reviews in Plant Sciences, 29(4), 204–221.

[ece35133-bib-0046] Zhang, D. S. (2009). Sandy desertification and its control in the Qinghai Plateau (pp. 6–40). Beijing, China: Science Press.

[ece35133-bib-0047] Zhao, X. C. , Lai, L. M. , Zhu, L. H. , Wang, J. J. , Wang, Y. J. , Zhou, J. H. , … Zheng, Y. R. (2014). Fine root biomass, decomposition and turnover of *Reaumuria soongorica* communities in the Sangong River basin. Acta Ecology Sinica, 34(15), 4295–4303.

